# No sound is more distracting than the one you're trying not to hear: delayed costs of mental control of task-irrelevant neutral and emotional sounds

**DOI:** 10.1186/s40359-022-00751-6

**Published:** 2022-02-21

**Authors:** Örn Kolbeinsson, Erkin Asutay, Manja Enström, Jonas Sand, Hugo Hesser

**Affiliations:** 1 Department of Behavioural Sciences and Learning, Linköping University, Hus I:3, 581 83 Linköping, Sweden; 2 School of Law, Psychology and Social Work, Center for Health and Medical Psychology, Örebro University, Örebro, Sweden

**Keywords:** Sound, Mental control, Suppression, Emotion

## Abstract

**Background:**

Suppressing intrusive thoughts can result in a post-suppression rebound effect where the same thoughts become hyperaccessible. The current study aimed to investigate if similar so-called rebound effects could be observed when people attempted to mentally suppress awareness of nonsensical auditory stimuli. Based on previous research on thought suppression and mental control in other domains, we hypothesized that attempting to suppress awareness of a task-irrelevant sound while under cognitive load would impact evaluations of the sound on affective dimensions and loudness, and result in increased general vigilance, as evidenced by faster responding on subsequent tasks.

**Methods:**

We performed two experiments where participants in a suppression condition were initially instructed to mentally suppress awareness of a sound while performing a mentally challenging task. Participants in a control condition performed the same task without receiving any instructions regarding the sound. In Experiment 1, the sound was affectively neutral, while in Experiment 2 participants were presented with an inherently aversive (tinnitus-like) sound. After this initial phase, participants performed tasks measuring vigilance and attention, and were also asked to give subjective ratings of the sounds on a number of affective dimensions and loudness.

**Results:**

In Experiment 1, participants in the suppression condition showed faster response times on both a visual search task and an auditory spatial cueing task, as compared to participants in the control condition. Contrary to our predictions, participants in the suppression condition did not rate the distractor sound as louder than participants in the control condition, and there were no differences on affective dimensions. In Experiment 2, results revealed that participants in the suppression condition made more errors on a visual search task, specifically on trials where the previously suppressed sound was presented. In contrast to results from Experiment 1, participants in the suppression condition also rated the targeted sound as louder.

**Conclusions:**

The findings provide preliminary support for a post-suppression rebound effect in the auditory domain and further suggest that this effect may be moderated by the emotional properties of the auditory stimulus.

## Background

An irrelevant sound can compel our attention: it can disrupt ongoing thought processes and mental activity. This is especially true for emotionally significant sounds [1, 2] as they increase our vigilance and are capable of redirecting our focus [3]. A common strategy when distracted by sound, emotional or otherwise, is to limit exposure to the sound by, for example, using noise-cancelling headphones or moving away from the sound source [4]. However, physical avoidance strategies are not always possible and can, in many cases, be impractical. Attempting to divert attention away from the distracting stimulus is one strategy to sustain goal-directed activities or reduce discomfort when faced with an unavoidable, disrupting sound. It is also reasonable to assume that the degree to which the individual can block or eliminate awareness of the sound is proportional to the degree to which they can control their thought processes and reduce distraction and discomfort. However, previous research on attempts to control mental states suggests that such attempts seldom yield the desired results. In fact, just the opposite tends to occur, as noted by Wegner in his Ironic Processes theory of mental control [5]. Wegner posits that attempts at mental control, that is attempting to alter or eliminate mental states such as thoughts, sensations, and perceptions, will fail in the long run and result in the opposite of what was intended. For instance, engaging in thought suppression can lead to the frequent intrusive return of the target thought over time [6, 7]. This *rebound effect* of thought suppression is particularly prominent if the suppressed material is emotionally significant [8, 9]. The current study examined whether these delayed consequences of thought suppression have an analogue in coping with task-irrelevant, distracting sounds.

As alluded to earlier, emotional stimuli will attract attention to a greater degree than neutral stimuli, as evidenced, for example, by their ability to bias attention when used as cues in dot-probe tasks [10, 11]. While this is beneficial when the emotional stimulus carries informational value [12, 13], it can be detrimental when an emotional stimulus is irrelevant to the task at hand [3]. Studies have also found that the valence and arousal of an emotional distractor can modulate the effects on performance. Sussman and colleagues [14], for example, found that negative valence was associated with poor task-relevant attention only when the stimulus was also highly arousing. In contrast, a low-arousal negative stimulus was associated with performance benefits when compared to neutral and positive counterparts. Regarding emotional, task-irrelevant sounds, these have been shown to cause greater distraction than neutral sounds on tasks requiring memory for serially presented material [1, 2], but have been shown to facilitate performance on tasks requiring quick responding [3]. These results have been interpreted as an effect of increased general vigilance and arousal in response to the emotional stimulus, which may offset the costs of reorienting attention toward the emotional sound [3, 15].

To cope with an emotional, distracting stimulus, some people will naturally attempt to put it out of their minds, not think about it, and suppress it from awareness. Suppression should, ostensibly, reduce the impact of the unwanted stimulus and allow one to engage in more productive activities. However, early studies by Wegner and colleagues [7, 9, 16] indicated that while suppressing unwanted thoughts may reduce their frequency in the short term, it has counterproductive long-term consequences. Wegner and colleagues [7] showed, for instance, that suppressing thoughts about a white bear caused those same thoughts to return with greater force when they were no longer being suppressed. Moreover, subsequent studies have indicated that if participants are forced to perform an unrelated mentally challenging task while suppressing a thought, suppression is more likely to fail [17, 18]. This has been interpreted to suggest that cognitive load, understood as a limit on available cognitive capacity imposed by simultaneously performing an unrelated task [19], interferes with the processes required for successful suppression.

Wegner [5] developed the Ironic Processes theory to explain the underlying processes of thought suppression and its paradoxical consequences. Wegner argued that suppression involves two simultaneous processes: the *Operating Process* and the *Monitoring Process*. The operating process attempts to reach the desired mental state—where the mind is free of the unwanted thought—by searching for, and focusing on, unrelated content. The monitoring process functions to verify that we are free of the unwanted thought by scanning for it and vigilantly reporting on any instances of failure. In this way, the monitoring process, which by necessity includes the material being suppressed, is mainly responsible for the rebound of suppressed material. The delayed consequences of suppression occur when the operating process stops filling the mind with unrelated content. Wegner argued that this results in hypervigilance towards the previously suppressed content, causing it to occur with greater frequency. Ample research on thought suppression has established that, under the right circumstances, thought suppression can be effective in the short term but produces paradoxical effects in the long term [20]. While early studies on thought suppression focused on occurrences of a target thought as the primary outcome, effects have since been found for several different outcomes, such as memory [21], mood [22] and decision making [23]. In addition to suppressing thoughts, suppressing emotions has also been identified as a potentially maladaptive regulation strategy. For example, Gross and John [24] found that people who use suppression to regulate their emotions tend to experience more negative emotion, show worse interpersonal functioning, and report lower levels of overall well-being. More generally, suppression has been implicated as an important factor in several psychopathological processes, such as OCD [25], PTSD [26], depression [27]. Most relevant for the current investigation, avoidance coping, and suppression especially, has also been implicated as detrimental coping strategies for dealing with tinnitus—the experience of sounds in the absence of an appropriate external sound source [28, 29].

While there is a lack of research on how mental coping strategies can impact disruption by auditory stimuli, there are findings that speak to the importance of attentional strategies in general, and suppression in particular, in the auditory domain [30]. Hesser and colleagues [30] examined the effects of suppressing awareness of an affectively negative sound, mimicking the psychoacoustic features of tinnitus, among normal-hearing participants. Following experimental manipulation (suppression vs. no instruction), the effect on persistence behavior in a subsequent, cognitively demanding task was examined. In line with predictions, a suppression instruction resulted in delayed consequences in the form of performance detriments on a persistence task relative to a no-instruction control.

Based on these results, the aim of the current study was to examine whether the rebound effect of thought suppression has an analogue in the processing of irrelevant sounds. Specifically, we aimed to explore whether suppression, as a mental coping strategy, was associated with a rebound effect—a prominent reemergence of the suppressed experience—defined as the extent to which the suppressed target sound interferes with subsequent task performance, increases subjective ratings of loudness and induces negative emotion. We employed dual-task designs in which participants were instructed not to think about a distracting sound in an initial phase and subsequently performed an unrelated task while being presented with the same sound. As cognitive load has been shown to impact the short-term effectiveness of suppression, we included a challenging serial recall task in the suppression phase, where the sound served as an auditory distractor. The sound was not expected to, in and of itself, significantly disrupt performance, as previous research suggests that steady-state sounds, such as those used in the current experiments, do not generally affect serial recall [31, 32]. After the initial suppression phase, the same sound acted as a cue or distractor on a vigilance- or attention task. We predicted that suppression would cause hypervigilance towards the target sound, resulting in faster responding on vigilance tasks and impaired performance on measures of attention. As we were interested in the effects of a sounds' emotional properties, these were varied between experiments: in Experiment 1, we used sounds expected to be emotionally neutral, while in Experiment 2, we used tinnitus-like sounds shown to elicit negative emotion. We predicted that suppression would be more difficult for more emotionally aversive and intrusive sounds and that negative emotional sounds would potentiate the rebound effects of suppression, more specifically in the form of an *emotional rebound*.

## Experiment 1

The experiment was set up as a between-subjects design, comparing a condition where participants received suppression instructions with a no instruction control condition. We employed a three-phase design to investigate the immediate and delayed effects of suppression. In the first phase, participants in the suppression condition were instructed to suppress a sound heard in the background while performing a serial recall task. Participants in the control condition received no instructions regarding the sound. The serial recall task is challenging for most people and was therefore expected to induce significant cognitive load. In the second phase, we asked participants to perform a visual search task and presented the same sound on each trial. As mental suppression tends to cause suppressed material to become hyperaccessible [7, 17], and previous studies have indicated that hypervigilance towards a stimulus can bias attention and cause faster responding [3, 14], we hypothesized that participants in the suppression condition would respond faster in the presence of the previously suppressed sound. Finally, participants completed an auditory spatial cueing task. We similarly predicted that participants in the suppression condition would respond faster on trials where the previously suppressed sound served as a cue. We also hypothesized that participants in the suppression condition would have greater difficulty disengaging attention from the distractor sound as compared to participants in the control condition. Finally, we asked participants about their subjective experience of the sound, and we hypothesized that the suppression manipulation would result in participants reporting greater negative valence, greater arousal, and more annoyance in response to the sound, and rating the sound as being louder, as compared to participants in the no instruction control condition.

### Methods

We report how we determined our sample sizes, all data exclusions (if any), all manipulations, and all measures in the study.

#### Participants and design

Sixty participants (34 female) were recruited from a university campus. Ages ranged between 19 and 45 (*M* = 23.7, *SD* = 6.11). Eligibility criteria for the study were: adequate proficiency in Swedish, normal or corrected-to-normal vision, no known hearing impairments, and no known issues concerning tinnitus or hyperacusis. All participants were compensated for their time with a small gift worth a maximum of SEK 100 (approximately 10€). Participants were randomly assigned to either the suppression condition or the control condition, with 30 participants in each group. Participants were evaluated on four outcomes: serial recall performance, visual search performance, auditory search performance and subjective ratings of the sounds.

## Materials

### Sound stimuli

The main sound used in the experiment was a broadband pink noise. The control sound, used in the auditory spatial cueing task and to compare subjective sound ratings, was a broadband white noise. All sounds were generated using Audacity software v. 2.4.2. (General Public License) and presented binaurally through a set of Beyerdynamic DT 770 headphones at a level of 65 dB(A).

#### Suppression manipulation

The main manipulation consisted of additional instructions provided to participants in the suppression condition prior to the serial recall task. After reading the standard serial recall instructions, the suppression group received the following additional instructions:

Your task is to, with all your power, shut out the sound you will hear through the headphones at the same time as you perform the recall task. We know that it can be difficult but really try hard not to think about the sound. If you still notice it, block it out as quickly as you can. Suppress and repress it. Distract yourself. Fight against it and take control.

The control group simply received the standard serial recall instructions without mention of the sound or any mental strategies.

#### Serial recall task

On the serial recall task, participants were presented with and asked to remember a sequence of seven consonants. On each trial, seven letters from the set {R, W, X, T, G, Z, C} were sampled without replacement and presented for 800 ms with an inter-stimulus interval of 200 ms. When the final letter had been presented, a text box appeared on the screen and participants were requested to input the first letter they had seen on the current trial and then wait for the next box to appear. This process continued until seven boxes, each representing one of the seven letters, had appeared. Participants completed five trials of serial recall. During the task, an auditory distractor was presented in the form of a broadband pink noise. Noise onset was 5 s before presentation of the first letter and persisted continuously until the participants had completed all five trials. Answers were scored according to a strict serial recall criterion, meaning that a letter had to be indicated at the correct serial position to be scored as correct.

#### Visual search task

In the visual search task, participants were instructed to search a circular array of visually presented symbols for a target symbol and then indicate the target symbol's orientation (left or right) using one of two buttons on the keyboard. Participants were instructed to perform this task as fast as possible without making any errors. They were also presented with three practice trials before the testing phase, where they received feedback on whether their response was correct or incorrect. No feedback was given on subsequent test trials. The participants completed 48 test trials in total. There were two levels of task difficulty, such that the arrays contained either 7 (easy) or 11 (hard) distractor symbols, in addition to the target symbol. Each trial was randomly selected to be either easy or hard, with the constraint that there would be 24 trials of each difficulty. Trials began with a fixation cross, presented for 2–4 s on each trial to minimize anticipation effects. The visual array and the auditory distractor were presented immediately after the offset of the fixation cross. The distractor was the same pink noise that had served as the distractor on the serial recall task.

#### Auditory spatial cueing task

Participants performed an auditory spatial cueing task in which they indicated the location (left or right) of an auditory probe. The probe was presented immediately after the offset of a 2000 ms duration cue. The cue was presented either in the same headphone as the probe or in the opposite headphone. The cue was either the previously heard auditory distractor or the control sound. The probe was a 1 khZ sine-wave tone with a duration of 50 ms. Trials on which the probe is presented in the same headphone as the previously heard cue are termed *valid* trials. Conversely, trials on which the probe is presented in the opposite headphone are termed *invalid* trials. Participants performed 64 trials of the auditory search task, but due to a coding error, only data from 63 trials were recorded. Similar procedures have long been used with visual stimuli to study visual attention [33], and more recently to study attentional bias toward emotional stimuli [34, 35].

#### Subjective ratings of the sounds

In order to assess the effects of the manipulation on participants subjective perceptions of the sound, participants were asked to rate the auditory distractor, along with a control sound (white noise) on dimensions of arousal (calming vs. highly arousing), valence (extremely negative vs. extremely positive), annoyance (not at all vs. extremely annoying) and loudness (hardly audible vs. extremely loud). Ratings were collected using a visual analogue scale. Participants used the computer mouse to drag a marker along a continuum anchored at 0 and 100, placing the marker at a location representing their experience of the sound. In addition to rating their experience of the auditory distractor (broadband pink noise), participants also rated their experience of a control sound (broadband white noise). The control sound was included to ascertain whether observed effects were specific to the sound or due to general factors such as mood or fatigue.

### Procedure

Participants performed the experiment individually in a sound-attenuated room. An experimenter initially introduced the procedural details by giving verbal information about the experiment. Subsequently, they were asked to sit in front of the experimental computer and to start the experiment when ready. In the first part of the experiment, participants were asked to respond to demographic questions and were subsequently introduced to the serial recall task. Participants received instructions that they should memorize the letters and would later need to recall them in order. After receiving instructions for the task, participants in the suppression condition received additional instructions to suppress the sound during the task (see “Materials”). In contrast, the control group received no further instructions. Participants then performed five trials of serial recall. After completing the serial recall task, participants received instructions for the visual search task and subsequently completed the practice trials. Instructions were identical across groups. Participants then completed 48 trials of the visual search task. Finally, participants were asked to rate the experimental sound and a control sound on dimensions of valence, arousal, annoyance, and loudness. The experiment lasted approximately 30 min in total.

### Data analysis and sample size determination

All statistical analyses were performed using R statistical software [36]. ANOVAs were conducted using the afex package [37]. Linear mixed-effects models were fit using the nlme package [38], and bootstrapped samples were drawn using the 'lmeresampler' package [39]. For both reported experiments, a minimum of 30 participants for each condition was deemed appropriate considering stability of parameter estimates and statistical power [40]. Using G-Power software [41], it was determined that at least 26 participants for each condition would be needed in order to detect a moderate interaction effect (${\widehat{\eta }}_{p}^{2}$ = 0.0385) with adequate power (0.8) with a 2 by 2 mixed design (correlation of 0.5). No a priori power calculation was performed specifically for linear mixed effects models.

#### Serial recall and sound ratings

Serial recall data were analyzed by calculating the proportion of correct responses and comparing groups (suppression vs. control) over trials using a mixed-design ANOVA. Sound ratings were also analyzed using four mixed-design ANOVAs, one for each dimension (valence, arousal, annoyance, and loudness). Suppression condition was the between-subjects variable, and sound (distractor vs. control sound) was the within-subjects variable. Given observed deviations from normality for certain variables, we additionally conducted robust ANOVAs with 20% trimmed means [42]. The results were then compared with the original analyses. If these analyses produced qualitatively the same conclusions, we only present results from the traditional ANOVA models.

#### Visual search- and auditory spatial cueing tasks

Response times (RTs) from visual- and auditory search tasks were analyzed using linear mixed-effect models (LMM [43]). Compared to analyzing RTs aggregated across trials using traditional data analytical models, LMMs have demonstrated advantages by offering a more flexible approach to handling dependence among observations due to both subjects and experimental stimuli [43, 44]. They additionally allow for a more fine-grained analysis of change across trials based on individual responses. Presently, model building followed recent recommendations for using LMM in experimental psychology [45]. Only RTs from correct responses were included in the analyses. Based on recommendations from Lachaud and Renaud [46], extreme observations were eliminated by calculating subject-specific means and standard deviation for each type of trial and then removing observations that were more than $\pm$ 3*SD* from the subject mean. For the visual search task, this meant that by-subject means and standard deviations were calculated for each level of difficulty (Easy vs. Hard). On the auditory spatial cueing tasks, they were calculated separately depending on trial validity (valid vs. invalid). In total, 96 observations were eliminated from the visual search task (3.33% of total trials; 88 errors and 8 extreme observations), and 42 trials were eliminated from the spatial cueing task (1.67% of total trials; 10 errors and 32 extreme observations). Based on the experimental design, the LMM for visual search RTs included fixed effects of suppression condition (suppression vs control), trial, and difficulty level (easy vs. hard). The model for RT on auditory search performance trials, included fixed effects for suppression condition, trial, sound (distractor vs. control), and validity (valid vs. invalid trial). Additionally, the three-way interaction between suppression condition, sound, and validity, and all associated two-way interactions, were included as fixed effects in the model. In accordance with recommendations from Barr and colleagues [47], models were initially fit using a maximal random effects structure including subject-specific random effects for all within-subject variables, with an associated unstructured covariance structure. If the initial models did not converge, they were simplified by removing random effects that did not significantly contribute to the model, as determined by likelihood ratio tests. Inferential tests for fixed effects parameters in the linear mixed models were conducted using non-parametric confidence intervals obtained from (residual) bootstrapping with 3000 samples drawn with replacement [48]. Bootstrapping is less dependent on asymptotic distributional assumptions of maximum likelihood estimation in LMM, and even when assumptions are violated, resampling methods can be used to obtain consistent estimators of standard errors [48].

### Results

#### Serial recall

Serial recall performance did not differ between the suppression group (*M* = 0.55, *SD* = 0.29) and the control group (*M* = 0.57, *SD* = 0.29), *F*(1, 58) = 0.11, *p* = 0.739, ${\widehat{\eta }}_{p}^{2}$ = 002. There was a main effect of trial, *F*(2.92, 169.26) = 4.38, *p* = 0.006, ${\widehat{\eta }}_{p}^{2}$ = 0.070, indicating that participants improved over time. There was no significant interaction between condition and trial, *F*(2.92, 169.26) = 0.48, *p* = 0.689, ${\widehat{\eta }}_{p}^{2}$ = 008.

#### Visual search performance

Results from the LMM showed a significant effect of suppression condition, *b* = − 51.13, 95% CI [− 73.10, − 24.36], indicating response times were on average 51 ms faster for participants in the suppression condition as compared with controls. Furthermore, there was a significant effect of difficulty, *b* = 236.68, 95% CI [216.75, 264.99], reflecting longer RTs on the more difficult trials, and an effect of trial, *b* = − 3.33, 95% CI [− 4.65, − 2.88], indicating faster responding on later trials.^1^

#### Auditory spatial cueing task

Similar to results from the visual search task, results from the auditory spatial cueing task revealed a main effect of suppression condition, *b* = − 10.07, 95% CI [− 14.69, − 5.49], once again suggesting faster RTs for the suppression group. Moreover, responses were faster on invalid trials as compared to valid trials, *b* = 23.45, 95% CI [18.73, 28.01] and in response to the distractor sound as compared to the control sound, *b* = − 6.08, 95% CI [− 10.70, − 1.46]. Finally, participants were faster on later trials compared to earlier trials, *b* = − 0.56, 95% CI [− 0.69, − 0.44]. No interactions reached statistical significance.^2^

#### Subjective sound ratings

Means and standard deviations for all sound ratings across the two conditions are presented in Table 1.

**Table 1:** Descriptive statistics for subjective sound ratings from Experiment 1

Sound	Dimension	Suppression condition		Control condition	
		M	SD	M	SD
Distractor					
	Annoy	47.03	32.97	60.03	21.34
	Arousal	43.38	29.10	52.40	25.26
	Loud	49.76	26.26	65.70	15.04
	Valence	37.78	20.36	37.41	22.23
Control					
	Annoy	60.16	23.93	62.46	20.00
	Arousal	54.85	28.06	56.95	20.69
	Loud	55.78	15.33	57.22	13.91
	Valence	32.54	21.40	33.45	22.00

Subjective sound ratings rated on a VAS-scale between 0 and 100. Higher ratings indicate greater annoyance, arousal and loudness, and more positive affective valence

##### Annoyance

The 2 by 2 ANOVA for annoyance showed no significant main effect of condition, *F* (1, 38) = 1.24, *p* = 0.272, ${\widehat{\eta }}_{p}^{2}$ = 0.032, and no significant main effect of sound, *F*(1, 38) = 3.85, *p* = 0.057, ${\widehat{\eta }}_{p}^{2}$ = 0.092. Finally, there was no significant interaction between condition and sound, *F*(1, 38) = 1.82, *p* = 0.186, ${\widehat{\eta }}_{p}^{2}$ = 0.046.

##### Arousal

The ANOVA for arousal showed no significant main effect of condition, *F*(1, 38) = 0.67, *p* = 0.420, ${\widehat{\eta }}_{p}^{2}$ = 0.017, and no main effect of sound, *F*(1, 38) = 3.04, *p* = 0.089, ${\widehat{\eta }}_{p}^{2}$ = 0.074. The interaction between condition and sound was also non-significant, *F*(1, 38) = 0.57, *p* = 0.456, ${\widehat{\eta }}_{p}^{2}$ = 0.015.

##### Valence

The ANOVA for valence ratings indicated no significant main effects or interactions, all *F*’s < 2.4, all *p*’s > 0.13.^3^

##### Loudness

For ratings of loudness, the main effects of suppression condition *F*(1, 38) = 2.89, *p* = 0.097, ${\widehat{\eta }}_{p}^{2}$ = 0.071, and sound condition, *F*(1, 38) = 0.20, *p* = 0.656, ${\widehat{\eta }}_{p}^{2}$ = 0.005, were statistically non-significant. The interaction effect between sound and condition was statistically significant, *F*(1, 38) = 7.01, *p* = 0.012, ${\widehat{\eta }}_{p}^{2}$ = 0.156.^4^ None of the post-hoc tests revealed statistically significant differences when adjusting for multiple comparisons.

### Discussion experiment 1

In line with predictions, results from Experiment 1 suggest a delayed effect of mentally suppressing awareness of a task-irrelevant sound. While there was no indication of an initial effect, as evidenced by the lack of difference between groups on the serial recall task, results from the visual search- and auditory cueing tasks indicate a post-suppression rebound effect. Participants in the suppression condition responded faster on both tasks, suggesting the manipulation may have had a general effect on participants' vigilance. However, we were unable to detect any significant interactions in the auditory spatial cueing task, which would have indicated specific effects on attention related to the target sound. There was also no effect on subjective ratings of annoyance, valence or arousal, suggesting that the manipulation did not impact affective stimulus evaluation. Most importantly, the significant interaction between sound and suppression condition on loudness ratings revealed that the manipulation influenced loudness perception. However, while none of the post-hoc tests revealed statistically significant differences, the descriptive statistics show that the direction of the effect was unexpected, as participants in the suppression condition rated the distractor as less loud than participants in the control condition.

## Experiment 2

Emotional salience, especially threat value, has been shown to increase a stimulus' ability to capture and hold attention [49] and modulate the effects of thought suppression [9]. In Experiment 2, we used the same paradigm as in Experiment 1 to investigate the immediate and delayed effects of suppressing tinnitus-like sounds, which have previously been shown to be inherently aversive [30, 50].

### Methods

#### Participants and design

In total, 81 individuals participated in the experiment. One participant was excluded subsequent to participation due to a failure to complete the experimental tasks. The final sample thus consisted of 80 participants (50 female) aged between 18 and 40 years old (*M* = 23.3, *SD* = 3.9). Inclusion criteria were the same as for Experiment 1. Participants were randomly assigned to either the suppression (41 participants) or control condition (39 participants) and were evaluated on four outcomes: serial recall performance, visual search performance, auditory search performance, and subjective ratings of the sounds.

#### Materials

##### Sound stimuli

Two sound stimuli were presented in the experiment. The first sound (Sound 1) was a pure sine wave tone with a frequency of 4.20 kHz, while the second sound (Sound 2) was an amplitude modulated sine wave tone with a frequency of 4.56 kHz. Both sounds were generated using an AD229e diagnostic audiometer and presented binaurally through a set of Telephonics TDH-39P headphones at a level of 65 dB(A).

##### Manipulation

Participants in the suppression conditions were presented with the following instructions:

You will hear a sound through the headphones. You should mentally suppress this sound any way you can as long as you can hear the sound. Try as hard as you can, at the same time as you perform the task with the letters, to mentally suppress, fight against, shut off, distract yourself, or in some other way mentally avoid the sound. It may be difficult, but try as hard as you can to suppress the sound at the same time as you perform the task. IT IS VERY IMPORTANT THAT YOU DO ALL THAT YOU CAN TO SUPPRESS THE SOUND THROUGHOUT THE WHOLE TASK.

The no instruction control group simply received standard serial recall instructions with no mention of the sound or any particular mental strategies.

##### Serial recall task

The serial recall task was similar to that in the previously described studies, with some minor differences. The task consisted of one practice trial and seven test trials on which participants were asked to recall and input seven serially presented letters. The letters were randomly sampled from the set {R, H, L, K, F, M, Q} without replacement and presented immediately after one another with a duration of 1000 ms. One of the sounds (Sound 1 or Sound 2), counterbalanced across participants and conditions, was presented continuously throughout the task and served as an auditory distractor.

##### Visual search task

The visual search task used in this experiment differed substantially from those used in the previously described experiments. The task was based on those described by Treisman and Gelade [51] and consisted of three blocks of 6 trials. For all blocks, participants were presented with Os, Ns, or Xs in green, blue or brown, randomly dispersed on a white background. In the first block, participants were asked to identify whether one of the letters was blue, and indicate their answer by pressing one of two buttons for either yes or no. In the second block, participants were asked to identify whether one of the letters was either a brown or a green O. In the final block, participants were to identify whether one of the letters was either a green O or a green N. Before each trial, participants were presented with one of the two sounds for a duration of 8000 ms. The offset of the auditory stimuli was simultaneous to the onset of the visual stimuli involved in the search task. Each sound was presented on three consecutive trials during each block, followed by three consecutive trials of the other sound. The order of the sounds was counterbalanced across conditions and participants.

##### Self-report measures

Participants were asked to rate the two sounds on measures of valence and arousal using the Self-Assessment Manikin (SAM [52]). The SAM is an affective rating system consisting of five pictures for each scale representing five levels of valence or arousal. The participant can select any of the five pictures or a point halfway between two pictures, resulting in a 9-point scale. Further, participants were asked to indicate how loud they perceived the sound to be on a scale of 1 (barely audible) to 100 (extremely loud), and how annoying they found the sound between 1 (not at all) and 100 (extremely annoying). Finally, we added a measure of compliance by asking how well participants had followed instructions throughout the experiment on a scale of 1 (not at all) to 5 (entirely).

#### Procedure

Experimental procedures were similar to those in the previous experiment. After receiving verbal information about the experiments, participants were seated at the experimental computer and asked to begin the experiment when they were ready. In the first phase of the experiment, participants performed the serial recall task. They first completed a practice trial in silence, and then seven trials in the presence of one of the auditory distractors, counterbalanced across participants and across experimental conditions. Participants in the suppression condition received the suppression instruction prior to the practice trial. The remaining participants simply received no instructions regarding the sound. In the second phase of the experiment, participants completed a visual search task with the two sounds figuring as auditory distractors. Participants first completed one practice trial in silence and then completed three blocks of 6 trials, each with a different target condition. Each of the sounds was presented on three consecutive trials in each block. The order of the sounds was counterbalanced across participants and conditions. Finally, participants completed the self-report measures related to the sounds and to their psychological state. The experiment lasted between 15–25 min, depending on participants' individual capabilities.

#### Data analysis

Serial recall performance was analyzed using a mixed models ANOVA. Suppression condition (suppression vs. control) was included as a between-subjects factor, and trials were included as repeated measures. Sound ratings were also analyzed using mixed design, where suppression condition (control vs. suppression) was the between-subjects factor and sound (distractor vs. control) was included as a within-subjects variable. Robust versions of the ANOVAs were also conducted but are only reported if the results are qualitatively different from the traditional models. Response times from the visual search task were analyzed using a linear mixed-effects model, following similar fitting procedures as in Experiment 1. Only RTs from correct responses were included. As participants did not complete enough trials to enable a by-subject analysis of extreme observations, this was done only by difficulty. Specifically, means and standard deviations for each difficulty level were calculated, and observations 3*SD* removed from the mean were eliminated. This resulted in the elimination of 20 additional observations. Finally, one observation was removed as the RT was below 200 ms, which was deemed implausible. The model included fixed effects for suppression condition (suppression vs. control) as a between-subjects factor, sound (distractor vs. control), difficulty (easy vs. medium vs. hard), and trial. Accuracy on the visual search task was analyzed using a generalized linear mixed model (GLMM) for a binary outcome using a logit link. The fixed effects were the same as for the RT LMM, and the inclusion of random effects was determined using the same model building procedure (as documented in Experiment 1).

### Results

#### Serial recall task

The ANOVA for serial recall performance showed a main effect of trial, *F*(5.60, 437.11) = 3.15, *p* = 0.006, ${\widehat{\eta }}_{p}^{2}$ = 0.039 reflecting an improvement of serial recall performance over time. There was no significant effect of suppression condition or interaction between trial and suppression condition (*F*s < 0.7, *p*s > 0.41).

#### Visual search task

**Fig. 1:**
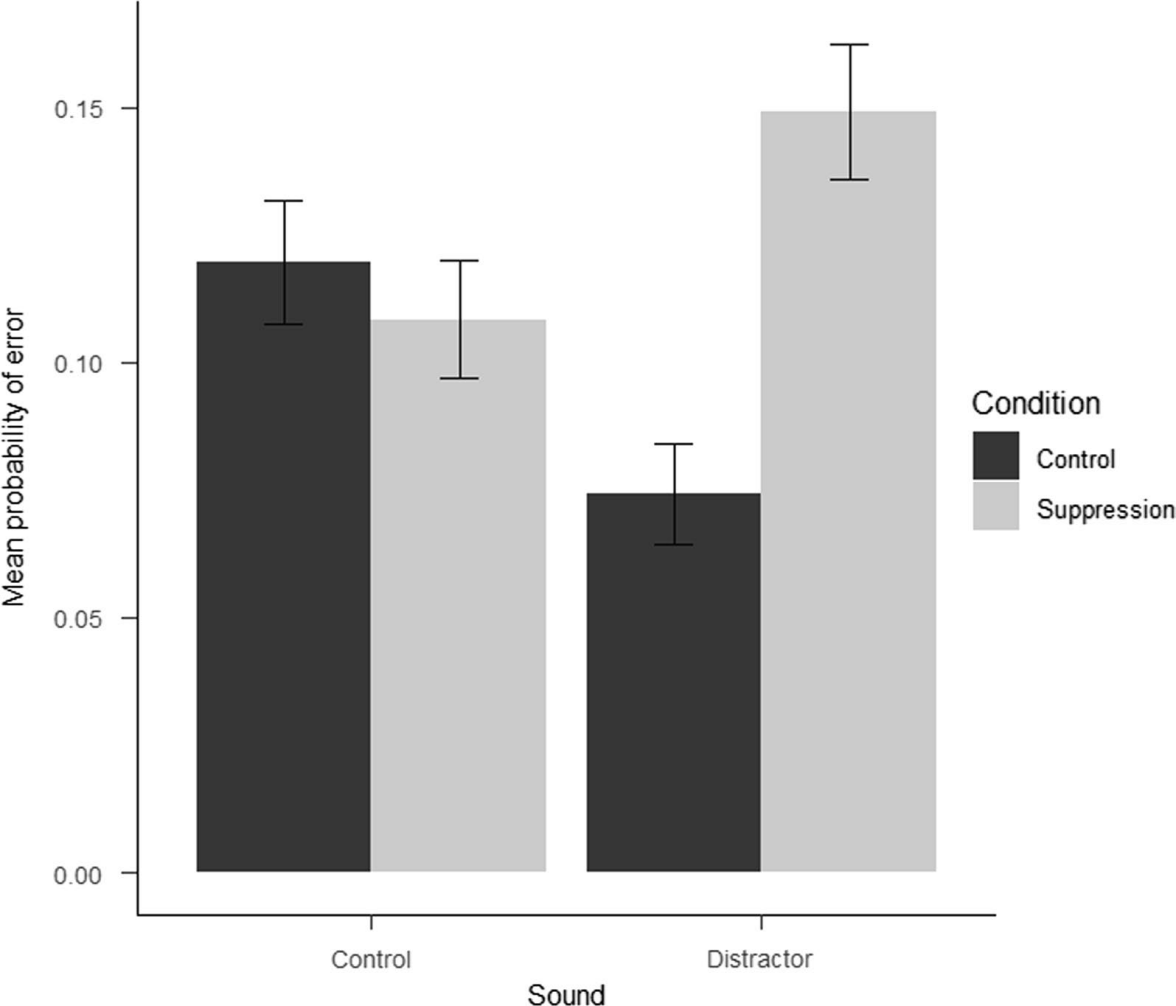
Mean observed probability of making an error, across individuals and trials, on the visual search task in Experiment 2

The main effects for trial, *b* = − 33.69, 95% CI [− 43.07, − 17.05], and difficulty, *b* = 430.93, 95% CI [364.72, 528.95] were significant, showing that participants responded more slowly on more difficult trials and that their performance improved over time. No other main or interaction effects reached statistical significance.^5^ A total of 163 errors (11.3% of total trials) were registered on the visual search task. The GLMM for accuracy showed a significant effect of difficulty, $\beta$ = 0.87, 95% CI [0.24, 1.49], *OR* = 2.38, indicating a higher probability of making errors on more difficult trials. Finally, there was a significant interaction between condition and sound, $\beta$ = 0.92, 95% CI [0.19, 1.64], *OR* = 2.50, showing that participants in the suppression condition were 2.50 times more likely to make an error on trials where they heard the distractor (see Fig. 1).

#### Subjective sound ratings

Means and standard deviations for all sound ratings across the two conditions are presented in Table 2.

**Table 2:** Descriptive statistics for subjective sound ratings from Experiment 2

Sound	Dimension	Suppression condition		Control condition	
		M	SD	M	SD
Distractor					
	Valence	4.18	1.39	4.39	1.15
	Arousal	5.84	1.74	6.06	1.81
	Annoyance	62.88	26.92	67.08	24.21
	Loudness	58.66	23.72	58.90	22.04
Control					
	Valence	4.31	1.88	4.41	1.44
	Arousal	5.53	1.75	5.67	2.15
	Annoyance	61.32	24.03	70.38	23.71
	Loudness	51.17	22.49	62.31	20.67

Valence and arousal were rated between 1 and 9 using the SAM. Annoyance and loudness were rated on a VAS-scale between 0 and 100. Higher ratings indicate positive valence, more arousal, more annoyance and more loudness

##### Annoyance

The ANOVA for annoyance ratings showed no main effects of either condition or sound and no interaction between the two (all *F*s < 1.69, all *p*s > 0.19).

##### Arousal

There were no significant main effects or interactions for ratings of arousal (*F*s < 2.46, *p*s > 0.12).

##### Valence

None of the main or interaction effects were significant for ratings of valence (*F*s < 0.47, *p*s > 0.49).

##### Loudness

There were no significant main effects of either suppression or sound on ratings of loudness. The interaction between the two was however statistically significant, *F*(1, 78) = 8.49, *p* = 0.005*,* ${\widehat{\eta }}_{p}^{2}$ = 0.098. Post-hoc tests revealed a significant difference between the distractor sound and the control sound in the suppression group, *t*(78) = − 2.87, *p* = 0.027, while there was no such difference in the control group, *t*(78) = 1.27, *p* = 0.583. Specifically, participants in the suppression condition rated the distractor sound as significantly louder than the control sound.

##### Compliance

Participants indicated high level of compliance with the instructions (*M* = 4.53, *SD* = 0.68). There were no differences between the conditions, *t*(76) = 1.01, *p* = 0.316.

### Discussion experiment 2

Results revealed no immediate effect of the suppression manipulation on serial recall performance, nor was there a delayed effect on RTs in the visual search task. However, consistent with the prediction of a post-suppression rebound effect, suppression was associated with increased error rates during visual search trials where the distractor was presented. While the manipulation did not impact sound ratings on affective dimensions, there was an effect on loudness ratings. In line with predictions, participants in the suppression condition rated the distractor sound, which had previously been targeted for suppression, as louder when compared to participants in the control condition.

## General discussion

We performed two experiments investigating the delayed effects of mentally suppressing awareness of a potentially distracting sound. Consistent with findings from the literature on thought suppression in other domains, we found evidence of a post-suppression rebound effect. In both experiments, we found between-group differences on visual search task performance: In Experiment 1, participants in the suppression condition responded faster on visual search- and auditory spatial cueing trials when compared to participants in the no-instruction control condition. In Experiment 2, while there was no effect on response times, we found that participants in the suppression condition made significantly more errors on trials where the sound previously targeted for suppression was presented. Taken together, these findings suggest that there are delayed effects of attempting to suppress awareness of task-irrelevant sound and add to the experimental literature on the post-suppression rebound effect, which has previously been observed in other domains.

Response times from the visual search- and auditory spatial cueing tasks suggested that the suppression manipulation influenced vigilance and attention. As pointed out by Max and colleagues [3], there are two ways in which a concurrent sound will impact response times: first, the sound may elicit an orienting response, potentially resulting in increased response times. Second, it may increase general arousal and vigilance, leading to faster responding. In experiment 1, participants in the suppression condition responded faster on visual search- and auditory spatial cueing trials compared to their counterparts in the control condition. These findings are similar to previous findings by Max and colleagues [3], which showed that negative sounds may be less detrimental to performance than neutral sounds on a cross-modal oddball task. The authors argue that any orienting costs resulting from the presentation of the negative sound are offset by the participant becoming more vigilant and thus responding faster. Furthermore, Asutay and Västfjäll [15] showed that auditory-induced arousal resulted in speeded responding on a visual search task. The abovementioned findings support an interpretation where the suppression manipulation in experiment 1 resulted in hypervigilance towards auditory cues on subsequent tasks, thus facilitating faster responding. In contrast, results from experiment 2 showed no differences between groups on response times, but participants in the suppression condition were more prone to error when presented with the previously suppressed sound. One critical difference between the two experiments was the characteristics of the auditory stimuli. The broadband noise in the first experiment has no inherent emotional properties, while the tinnitus-like, high-pitched tone of the second experiment has been shown to be perceived as negative and arousing [30, 50]. The results suggest that while suppression had delayed consequences in both experiments, stimulus characteristics may be an important moderator.

The suppression manipulation also influenced perceived sound intensity as measured by subjective loudness ratings. In line with our predictions, results from Experiment 2 suggest that participants in the suppression condition rated the distractor as louder. In Experiment 1, however, we found no such effect. If anything, observed differences were in the opposite direction of what was predicted. The difference between results may once again be related to the differing emotional characteristics of the sounds used in the two experiments. The impact of emotion on perceptual processing is well established [53]. Conditioned fear has, for example, been shown to facilitate auditory pitch discrimination, and, regarding loudness specifically, studies have shown that the emotional meaning will impact loudness perception [54, 55]. Furthermore, the emotional valence of a to-be-suppressed stimulus has been shown to impact the effects of suppression [9].Once again, our findings imply that the effects of suppressing awareness of an auditory stimulus may be moderated by the emotional salience of the stimulus being suppressed. Mentally suppressing awareness of an aversive and intrusive sound causes it to be perceived as louder, whereas suppressing a neutral sound has no such effect.

Concerning the affective dimensions of stimulus evaluation, we predicted that attempting to mentally suppress awareness of a sound would cause increased ratings of arousal, negative valence, and annoyance towards the targeted sound. In the current study, we hypothesized that the suppression instruction would cause the targeted sound to become hyperaccessible and therefore, ironically, be perceived as more intrusive. As a result, we expected these participants to experience more negative affect, subsequently evaluating the distractor sound as more negative, arousing, and annoying. Contrary to our predictions, the suppression manipulation did not impact ratings on any of these dimensions. Regarding arousal, a previous study found that faster responding on a visual search task (the same task as used in Experiment 1) was related to higher levels of sound-induced arousal [15]. In the current experiment, participants in the suppression condition responded faster than control participants and might therefore have been expected to give higher ratings of arousal. However, ratings were given in relation to the specific sounds. Results from the auditory spatial cueing task indicated that increased vigilance caused by the suppression manipulation was not specific to the sound that participants had been instructed to suppress from awareness. It is possible that participants in the suppression condition experienced a higher level of general arousal, which was not measured by the rating scales. More general questions regarding mood and arousal, along with physiological measures, would be useful in testing this interpretation.

In experiment 2, participants in the suppression condition were more likely to make errors in the presence of the distractor sound than participants in the control condition. One interpretation is that mentally suppressing awareness of the sound resulted in differential stimulus processing, leading the distractor to more readily capture attention after suppression. A possible way of accounting for this effect is through the functional view of auditory distraction proposed by Röer et al. [2]. The authors argue that task-irrelevant sound is processed simultaneously with focal stimuli in order to alert the organism when a motivationally relevant stimulus is detected. According to this view, voluntary attention may prevent conscious processing of irrelevant stimuli, but basic processing will nevertheless occur to monitor for important events. While allowing for detection of potential threat or reward cues in the environment, this openness of the auditory system renders it susceptible to distractors. Which stimuli will capture attention, that is which stimuli are considered motivationally relevant, is often determined by general aspects such as sudden changes in the environment [56], or emotional salience [3]. But there is also research suggesting that contextual factors, such as monetary incentives [12, 57], and mental states, such as hunger [58], can influence which stimuli are processed as motivationally relevant, and therefore capture attention. Viewed through the lens of the functional account of auditory distraction, our results suggest that attempting to mentally suppress awareness of emotional stimuli may enhance their motivational relevance.

One explanation for the detrimental effects of mental suppression may be that it serves an avoidance function. Thought suppression has, for instance, been studied as a form of cognitive avoidance, resulting in delayed effects on attention [59]. Studies on attentional avoidance of visual stimuli have also revealed ironic effects, where participants are more distracted by an irrelevant stimulus on a visual search task [60]. From a contextual behavioral perspective, suppression has been conceptualized as a form of experiential avoidance [61], which has been associated with a range of negative effects in a wide variety of circumstances [62]. For example, suppression has been shown to have paradoxical effects when attempting to reduce dysfunctional behavior such as overeating [63] and smoking [64]. Similarly, avoidance of fear-provoking stimuli can serve to immediately reduce discomfort, but will in certain circumstances have counterproductive long-term consequences in the form of, for example, maintaining debilitating fear [65]. Thus, the different stimulus characteristics of the sounds used in the two experiments may have resulted in the suppression behavior serving different functions and thus having different consequences. In experiment 1, the stimulus which participants were instructed to suppress was relatively neutral, and suppression is unlikely to have served a clear avoidance function. In experiment 2, however, the sound was inherently aversive, and suppression is therefore more likely to have been seen as a way to avoid the negative impact of the stimulus. Similar ideas have been proposed in the field of tinnitus [29, 66], where it has been suggested that avoidance coping may lead tinnitus to take on more frightening connotations, resulting in greater interference and distress over time. A growing amount of correlational research lends broad support to such notions, and psychological therapies that address maladaptive avoidance seem to be helpful for those suffering from distressing tinnitus [28, 67]. While our findings may only have indirect implications for tinnitus, understanding how stimulus characteristics alter the function served by mentally suppressing awareness of distracting sound may help us understand how suppression can be beneficial in certain circumstances while being maladaptive in others.

Several limitations must be considered when interpreting the results. First, both experiments employed a no-instruction control condition, allowing participants to use any mental strategies they naturally prefer, including suppression. Having an active control condition in which participants were given an alternative attentional strategy, such as focused attention or distraction, could have been an alternative. Second, the experiments did not include a manipulation check to confirm that participants in the suppression condition used suppression as an attentional strategy during the recall task. This limits our ability to draw firm conclusions on the specific effects of suppression, but it is difficult to devise a manipulation check to objectively determine the use of a mental strategy. While self-reported compliance ratings, such as those in experiment 2, may hint at whether participants managed to employ the strategy as intended, they come with their own set of problems, for example in the form of social desirability. Third, we only used artificial nonsense sounds in the form of broadband noise or modulated tinnitus-like tones. These sounds do not cause significant distraction on the serial recall task used in the suppression phase. Mentally suppressing awareness of more complex, inherently distracting sounds, is likely to be more challenging than suppressing simpler steady-state sounds such as those in the current study. Conducting similar studies with more complex and meaningful sounds is an important next step in replicating the current findings, or understanding how sound characteristics may moderate the effect of mental suppression. Nevertheless, showing ironic effects of suppression with meaningless, artificial sounds is an important first step in investigating the effects of suppression in the auditory domain. Fourth, while the performance tasks used in the current study were chosen to measure effects on attention and vigilance, other measures that are more similar to measures commonly used to study thought suppression could have been used. The most used paradigm in thought suppression research looks at a rebound in thought frequency after suspending suppression attempts [7]. While it is difficult to study the rebound of a sensation in awareness, we attempted to study whether an auditory stimulus becomes hyperaccessible to awareness after mental suppression. It is possible that other measures, such as perceptual discrimination tasks, are more sensitive to this kind of hyperaccessibility, and will therefore be better measures of an ironic rebound in the auditory domain. Further, we did not include any psychophysiological measures in our experiment. Including skin conductance measures would, for example, have allowed us to examine emotional responding in greater detail, such as arousal.

## Conclusions

While studies have previously investigated the effects of suppressing thoughts [7], suppressing pain sensation [68], and even suppressing awareness of visual stimuli [60], the auditory domain has been conspicuously neglected. Our study provides preliminary evidence that the delayed consequences of suppression, previously observed with other stimulus modalities, have an analogue in suppressing awareness of task-irrelevant sound. We showed that suppression impacted subsequent performance on visual search- and auditory cueing tasks and altered loudness perception. Our findings further suggest that the effects of suppression may be tied to the stimulus characteristics of the material which one attempts to suppress, and perhaps most importantly, to the emotional significance of said material. The findings may have implications for understanding psychological coping with regards to disruptive external sounds such as workplace noise and internally generated sounds such as tinnitus and emphasize the importance of continued research on the effects of attentional- and regulatory strategies on auditory distraction, emotion, and sound perception in general.

## Data Availability

All data, analysis code and materials are publicly available via the OSF repository, https://osf.io/hs72u/.

## Footnotes

1 To investigate potential habituation effects of repeated stimulus presentations we ran a post-hoc model including the condition by trial interaction. The interaction was not statistically significant, *b* = 0.29, 95% CI [− 1.79, 1.83].

2 A post-hoc model including the condition by trial interaction revealed no significant interaction, *b* = -0.10, 95% CI [− 0.35, 0.15].

3 The robust version indicated a significant main effect for sound, *p* = .035.

4 In the robust version, comparing 20% trimmed means, the main effect of condition was also statistically significant, *p* = .038, and the interaction effect remained significant, *p* = .020.

5 A post-hoc model including the condition by trial interaction revealed no significant interaction effect, *b* = − 5.78, 95% CI [− 14.99, 1.50].
